# A lifestyle intervention among elderly men on active surveillance for non-aggressive prostate cancer: a randomised feasibility study with whole-grain rye and exercise

**DOI:** 10.1186/s13063-016-1734-1

**Published:** 2017-01-13

**Authors:** Anne Kirstine Eriksen, Rikke Dalgaard Hansen, Michael Borre, Ryan Godsk Larsen, Jeppe Munthe Jensen, Kristian Overgaard, Mette Borre, Cecilie Kyrø, Rikard Landberg, Anja Olsen, Anne Tjønneland

**Affiliations:** 1 Unit of Diet, Genes and Environment, Danish Cancer Society Research Center, Strandboulevarden 49, 2100 Copenhagen, Denmark; 2 Institute for Clinical Medicine – Department of Urology, Aarhus University Hospital, Palle Juul-Jensens Boulevard 99, 8200 Aarhus N, Denmark; 3 Physical Activity and Performance Group, SMI, Department of Health Science and Technology, Aalborg University, Fredrik Bajers Vej 7, 9220 Aalborg, Denmark; 4 Section of Sport Science, Department of Public Health, Aarhus University, Dalgas Avenue 4, 8000 Aarhus, Denmark; 5 Department of Hepatology and Gastroenterology, Aarhus University Hospital, Nørrebrogade 44, 8000 Aarhus C, Denmark; 6 Food and Health, Department of Food Science, Swedish University of Agricultural Sciences, Almas Allé 5, 75007 Uppsala, Sweden; 7 Food and Nutrition Science, Department of Biology and Biotechnology, Chalmers University of Technology, SE-412 96, Gothenburg, Sweden; 8 Unit of Nutritional Epidemiology, Institute of Environmental Medicine, Karolinska Institute, SE-171 77, Stockholm, Sweden

**Keywords:** Whole-grain rye, Physical activity, Prostate cancer, Intervention, Feasibility

## Abstract

**Background:**

The prognosis for men with non-aggressive prostate cancer is good, and several studies have investigated the impact of lifestyle changes including physical activity and diet on the prognosis. Despite positive results in animal studies and a few human interventions with whole-grain rye on markers of prostate cancer progression, the feasibility of trials investigating such dietary changes in combination with physical activity remains largely unstudied. The primary aim was to investigate the feasibility of an intervention with high whole-grain rye intake and vigorous physical activity for 6 months in men diagnosed with prostate cancer.

**Methods:**

In total, 26 men (53–72 years) recently diagnosed with non-aggressive prostate cancer and on active surveillance, were enrolled in 2011–2012 and randomly assigned to an intervention or a control group. The intervention included 170 g/day of whole-grain rye and 3 × 45 minutes/week of vigorous physical activity. The duration of the intervention was 6 months and end of follow-up 12 months after baseline. Clinic visits were scheduled at baseline and 3, 6 and 12 months after baseline. Compliance with the intervention was evaluated by diaries, food frequency questionnaires, biomarkers, and heart rate monitor data. The effect of the intervention was evaluated by linear multiple regression analysis.

**Results:**

In the intervention group, the mean daily intake of whole-grain rye measured from diaries was 146 g (SD: 19) for the first 3 months and 125 g (SD: 40) for the last 3 months of the intervention. The median level (5^th^ and 95^th^ percentiles) of vigorous physical activity was 91 (17, 193) min/week for the first 3 months and 66 (13, 259) min/week for the last 3 months. No recordings of physical activity were done for the control group. Aerobic fitness (VO_2_ peak) increased in the intervention group compared to the control group after the intervention. No effects were found on other cardio-metabolic outcomes or prostate cancer progression.

**Conclusions:**

The lifestyle intervention appeared feasible for 6 months among Danish men and the results are encouraging for conducting full-scale studies, where the impact of whole-grain rye and vigorous physical activity on prostate cancer progression and metabolic parameters can be evaluated.

**Trial registration:**

ClinicalTrials.gov, NCT01300104. Registered on 18 February 2011.

## Background

Prostate cancer is the most commonly diagnosed cancer among men in the Western part of the world [1]. In Denmark, it is the most common cancer and the second most common cause of cancer death among the male population [2]. The incidence of prostate cancer, especially indolent prostate cancers, has increased dramatically after the introduction of the prostate-specific antigen (PSA) testing in the 1990s [3]. Despite generally good prognoses, prostate cancer treatments, such as hormone-, radiation- or chemotherapy and prostatectomy surgery, often lead to a number of side effects such as urinary and erectile dysfunction and loss of typical male characteristics [4]. Therefore, an alternative strategy of long-term observation has been introduced called *active surveillance*, where men with prostate cancer considered to be non-aggressive are followed regularly by monitoring PSA levels and prostate biopsies. However, men on active surveillance living with an untreated prostate cancer have been reported to experience distress, from the uncertainty of whether the disease will progress [5], and pressure from relatives to choose an active treatment instead [6]. The current study was initiated to investigate if active involvement of the patient through lifestyle changes with diet and exercise was feasible in a group of men with non-aggressive prostate cancer on active surveillance.

One of the specific foods that has been in focus is whole-grain rye. Whole-grain rye and rye bran have a high content of dietary fibre, lignans and a number of other compounds with anticipated health effects [7]. In a small 3-week pilot intervention study in Sweden, high intakes of rye bran increased tumour apoptosis in men with prostate cancer [8], and a 6-week randomised crossover study found that high intakes of whole-grain rye resulted in lowered concentrations of PSA, fasting glucose, insulin, urinary C-peptide, and C-reactive protein in men with early-stage prostate cancer [9]. Furthermore, in animal studies, whole-grain rye reduced early prostate tumour growth, reduced concentrations of PSA, and increased epithelial cell apoptosis [10–12]. Whole-grain rye is rich in alkylresorcinols, phenolic lipids exclusively found in the outer layer of whole-grain wheat and rye among commonly consumed foods [13] that have been validated as concentration biomarkers of whole-grain wheat and rye intake [14].

Physical activity has been suggested to improve prognosis [15], quality of life, and physical fitness and well-being [16] among men with prostate cancer. In observational studies, both amount and intensity of physical activity have been associated with better prognosis and survival among prostate cancer cases [17–19]. Vigorous physical activity, for at least 3 hours/week compared to less than 1 hour/week, was associated with 61% lower risk of prostate cancer-specific mortality in a US cohort study [17]. The feasibility of increasing physical activity in combination with dietary changes in men with prostate cancer has been investigated [20] – also currently in ongoing studies [21, 22]. In all these studies, dietary interventions included reduced fat intake and increased plant-based foods, whereas neither included rye in the diets.

The primary aim of the present study was to evaluate the feasibility of introducing a comprehensive lifestyle intervention, with vigorous exercise and high whole-grain rye intake for 6 months, in a group of men with non-aggressive prostate cancer. Second, the effect of the intervention on cardio-metabolic outcomes, and further PSA levels, was evaluated.

## Methods

In brief, 26 Danish men aged 53–72 years, recently diagnosed (maximum 24 months from baseline) with non-aggressive prostate cancer and on active surveillance, were enrolled continuously over a period from March 2011 to November 2012 from the Department of Urology, Aarhus University Hospital, Denmark (www.ClinicalTrials.gov, identification number NCT01300104). At the time of diagnosis or as part of regular active surveillance visits at the Department of Urology, prostate cancer patients were informed about the Nordic Lifestyle Intervention Trial on Prostate Cancer Progression (NILS) study. The number of participants was based on the predicted number that the study centre was able to recruit within the time provided, and that the sample size would be sufficient to test the recruitment, randomisation, intervention, follow-up processes, and participant drop-out. The inclusion and exclusion criteria are described in Table 1. Eligible participants were informed about the NILS study orally and in writing as recommended in the current guidelines of the National Committee on Health Research Ethics (Denmark). After the participants had given informed consent, they were randomly allocated by a computer-generated list of random numbers to an intervention group (17 men) or a control group receiving standard active surveillance care (9 men) in a 2:1 scheme. In practice, the study nurse entered the participant number into a computer whereafter a program output specified which group the participant was randomised to. Neither participants nor practitioners were blinded to the group allocation. The duration of the intervention was 6 months and end of follow-up was 12 months after baseline. All participants attended regular scheduled clinic visits at baseline and 3, 6 and 12 months after baseline to monitor prostate cancer progression and general health through laboratory assessments and for collection of biological samples (blood and 24-hour urine samples at all four time points, and prostate tissue samples at baseline and 6 months after baseline) for subsequent biomarker analyses. At baseline, all participants were given a pamphlet on the at that time current dietary recommendations “The Eight Danish Dietary Recommendations” [23]. These include to walk a minimum of 10,000 steps per day and to consume fish, foods rich in fibre such as fruit and vegetables, and whole-grain products in the everyday diet, and to reduce the intake of saturated fat by use of low-fat meat, soft margarines and vegetable oil.

**Table 1:** Inclusion and exclusion criteria for study participation in the NILS feasibility study

Inclusion criteria	Exclusion criteria
Biopsy-proven prostate cancer within 2 years prior to enrolment	Less than 10 years of life expectancy
PSA ≤ 10, Gleason score ≤ 6, clinical classification ≤ cT2a or PSA ≤ 10, Gleason score ≤ 7, clinical classification ≤ cT2a	Prior history of cancer, except for non-melanoma skin cancer, unless considered cured without signs of treatment failure for at least 5 years
Maximum 1/5 tumour-positive biopsy rate	Conditions or behaviours likely to affect the capability of participating fully in the intervention
On active surveillance (elected to forgo treatment)	Moderate to severe co-morbidity (kidney, liver, heart, or respiratory problems)
Level of testosterone normal in sera	Inflammatory bowel disease or physical handicaps
Above 55 and below 70 years of age^a^	Gluten intolerance

^a^Was adjusted to include the age span of 53–72 years

The NILS feasibility study was approved by the Regional Ethics Committees on Human Studies in Copenhagen and Aarhus, Denmark (H-1-2010-073) in September 2010 and by the Danish Data Protection Agency (2010-41-5520) in February 2011.

### The intervention group

Participants in the intervention group were prescribed to consume a minimum of 170 g whole-grain rye per day as part of their daily diet. A list of whole-grain rye products accessible at local groceries and stores and an electronic scale was provided to all participants in the intervention group. Photographs of the products were included with information on whole-grain rye content per serving (e.g. one slice of bread, one portion of cereal) and the weight/size in grams of one serving. A point system with whole-grain rye content for the different products was developed to ease the task of reaching 170 g of whole-grain rye per day. The participants bought their own food, but three products were offered for free; milled whole-grain rye for breakfast meals (rye porridge) and baking, whole-grain rye pasta, and an instant powder to make the Danish dessert or breakfast dish “*øllebrød*” (a porridge-like dish made of rye bread and non-alcoholic beer). It was recommended to consume as much of the milled whole-grain rye as possible. The products provided were all 100% whole-grain rye. The target of 170 g of whole-grain rye per day was based on *Dietary habits in Denmark 2003 to 2008* [24], where a mean intake of bread, rice, pasta and cereals in men aged 18–75 years was 245 g per day. The NILS study participants were in the older range (53–72 years), presumably with a lower energy intake and furthermore, rice was not included as no similar rye product exists. Therefore, 170 g was chosen as a realistic amount of whole-grain rye per day assuming that all bread, pasta and cereals were substituted with whole-grain rye products during the intervention period.

Additionally, the intervention group was prescribed to exercise at a target intensity of 70% of maximal heart rate for at least 45 minutes three times per week and encouraged to walk at least 10,000 steps every day. Steps per day were monitored by pedometers provided to the participants for daily use, however, these data are not included as the step counts were unreliable. The 3 × 45 minutes were chosen to get a sufficient level of physical activity to be able to see a significant health beneficial effect [25], and at the same time a realistic level for the men in order to ensure compliance. The participants were instructed to exercise according to customised endurance training programmes. The training sessions were non-supervised, but the subjects were instructed to monitor and upload their activity using a GPS/heart rate monitor provided at the beginning of the intervention. The participants were further instructed to register all physical activity in diaries during the intervention period. For help and support to improve and maintain the regimen of the intervention, individual counselling sessions were prescribed with a dietician and a sports physiologist at baseline, week 2 and 5, and 3 months and 6 months after baseline. Additionally, two informal evening get-togethers were held to give the participants the opportunity to meet and to receive information about the intervention. The participants’ spouses were encouraged to attend the regular clinical meetings, counselling sessions and evening get-togethers.

### The control group

The participants in the control group were advised to follow the “The Eight Danish Dietary Recommendations” and encouraged to walk 10,000 steps every day. The control group was included in the study to allow for a randomised design, but also to test whether it was possible to have an actual control group who maintained their habitual lifestyle despite not receiving any treatment. For a future larger-scale study, this consideration is important for estimating drop-out rates and other factors.

### Physical measurements

Participants were examined at the research clinic (baseline, 3, 6 and 12 months after baseline) after an overnight fast. At each clinic visit, blood samples were drawn (26 mL) and spun to separate into plasma, serum, erythrocytes and buffy coat. The samples were then stored at -80 °C. Anthropometric measurements and seated resting blood pressure were performed at each visit. At baseline and after 6 months, participants had ten transrectal ultrasound-guided random prostate needle biopsies taken. Four extra biopsies were taken for research analyses. As part of normal procedure to avoid sepsis, a dose of 2 × 500 mg of the antibiotic Ciproxin (ciprofloxacin) was given prior to and 6–8 hours after the biopsy was taken. Peak aerobic capacity, a measure of aerobic fitness (peak oxygen consumption per unit time [VO_2_ max]) was assessed using a maximal progressive stepwise cycle ergometer test, a respiratory gas exchange analyzer (AMIS 2001; Innovision, Odense, Denmark), and a heart rate monitor (Polar Electro Oy, Kempele, Finland).

### Laboratory analyses

Analysis of plasma lipids, serum insulin and plasma glucose concentrations were performed by established routine methods at the certified laboratory of the Department of Clinical Chemistry at Uppsala University Hospital, Sweden. Total PSA was analysed at the Biochemical Laboratory at Aarhus University Hospital, Denmark. For alkylresorcinols, five different homologues were analysed (C17:0. C19:0, C21:0, C23:0 and C25:0) in plasma by a rapid gas chromatography-mass spectrometry method [26]. This made it possible to use the C17:0/C21:0 ratio as a measure of whether whole-grain intake primarily originated from wheat or rye.

### Dietary assessment

Information on diet was collected at baseline, 6 months and 12 months after baseline using semi-quantitative food frequency questionnaires (FFQ). The questionnaire given at baseline was similar to the validated FFQ from the Danish Diet, Cancer and Health cohort [27]. The questionnaire included 92 different foods covering the average intake of the preceding 6 months. A rye-specific FFQ was constructed for the present study for the 6-month visit restricted to 16 questions on specific rye products in a format similar to the baseline and follow-up FFQ. At the 12-month visit, participants were given the baseline FFQ along with the 16 rye-specific questions. Furthermore, the intervention group was instructed to complete daily diaries of rye intake including type and amount of rye products consumed. FFQ data were evaluated for all participants, at baseline, 6 months, and 12 months from baseline. Additionally, for the intervention group, the completed rye diaries were evaluated from baseline to 3 months and from baseline to 6 months. The whole-grain rye intake was calculated by multiplying the percentage of whole grains in the product using information from the manufacturers of the products. For bread and crispbread intake, participants reported consumed slices, which were translated to grams using standard portion sizes of 25 g for half a slice of rye bread and 12 g for a slice of crispbread.

### Statistical methods

The statistical analyses were based on the participants who completed the 6-month intervention period only (as opposed to intention-to-treat). Baseline characteristics, reported whole-grain rye intake, physical activity diary and heart rate monitor data, alkylresorcinols concentrations, and all physical and cardio-metabolic outcomes, and PSA levels are presented as means with standard deviations (SD) for the intervention and control group separately.

The effect of the intervention was evaluated by a linear multiple regression analysis model. The difference between the groups’ mean changes from baseline to 6 months were then tested by the linear multiple regression model using least square means, adjusted for baseline level of the outcome variable. Furthermore, the same analyses were made to test for differences in compliance markers (plasma alkylresorcinols concentrations, and the alkylresorcinol C17:0/C21:0 ratio) between the groups.

Since the NILS study was set up as a feasibility study, no power calculations were made.

The statistical analyses were conducted using the procedure general linear model (GLM) in the SAS® statistical software, release 9.3 (SAS Institute, Cary, NC, USA).

## Results

**Fig. 1:**
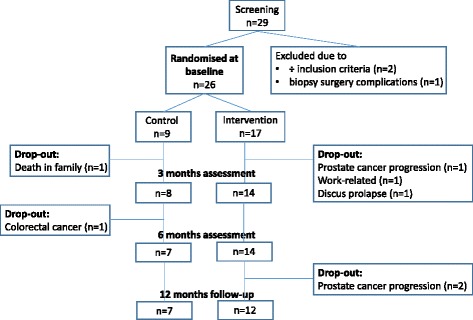
Flowchart of participants and drop-out in the NILS feasibility study

Twenty-one participants completed the 6-month intervention period; 14 in the intervention group and 7 in the control group (Fig. 1). Five participants dropped out due to prostate cancer progression (*n* = 1), work-related reasons (*n* = 1), discus prolapse (*n* = 1), death in the family (*n* = 1) and colorectal cancer (*n* = 1), of which three were from the intervention group and two from the control group. Further, two of the 14 participants in the intervention group dropped out before the 12-month examination (6 months after intervention ended) due to prostate cancer progression. Baseline characteristics of the participants, as shown in Table 2, were similar for the two groups.

**Table 2:** Baseline characteristics for intervention and control group – the NILS feasibility study

	Intervention (n = 14)	Control (n = 7)
	*Mean (SD)*	*Mean (SD)*
Age at baseline (y)	63 (5)	63 (5)
Age at PC diagnosis (y)	61 (6)	63 (6)
Weight (kg)	88 (17)	83 (10)
Height (m)	1.83 (0.06)	1.80 (0.04)
BMI (kg/m^2^)	26.0 (3.4)	25.6 (2.5)
Hip circumference (cm)	105 (10)	102 (6)
Waist circumference (cm)	102 (15)	98 (6)
Fat-free mass (kg)	65 (7)	64 (6)
Fat mass (%)	25 (6)	23 (4)
VO^2^ max (ml O^2^/min/kg)	29 (7)	26 (5)
Systolic BP (mmHg)	146 (19)	142 (13)
Diastolic BP (mmHg)	89 (10)	87 (8)
HDL cholesterol (mmol/L)	1.3 (0.2)	1.3 (0.2)
LDL cholesterol (mmol/L)	2.8 (0.7)	2.9 (0.6)
Total cholesterol (mmol/L)	5.1 (0.7)	5.3 (0.8)
Triglycerides (mmol/L)	1.3 (0.4)	1.5 (0.6)
Fasting plasma glucose (mmol/L)	5.8 (0.6)	6.1 (0.4)
Fasting blood glucose (mmol/L)	5.3 (0.5)	5.6 (0.5)
Blood glucose 2 h (mmol/L)	6.7 (2.2)	8.3 (2.0)
Fasting insulin (mmol/L)	11.6 (6.6)	11.8 (6.8)
Alkylresorcinols total (nmol/L)	109 (98)	74 (39)
C17:C21 alkylresorcinol ratio	0.39 (0.19)	0.30 (0.10)
Plasma enterolactone (nM)	51 (51)	52 (50)
Prostate-specific antigen (PSA) (ng/ml)	5.4 (1.7)	5.0 (2.2)

*SD* standard deviation, *PC* prostate cancer, *BMI* body mass index, *VO* ^*2*^ *max* peak oxygen consumption per unit time, *BP* blood pressure, *HDL* high-density lipoprotein, *LDL* low-density lipoprotein

### Whole-grain rye intake

Compliance with the whole-grain rye intervention diet was generally good, though slightly lower than the target of an average of 170 g of whole-grain rye per day (Table 3). In the intervention group, as reported in the daily rye diaries, the mean daily intake of whole-grain rye was 146 g (SD: 19) for the first 3 months and 125 g (SD: 40) for the last 3 months of the intervention. The whole-grain content of the rye products consumed in the intervention group ranged from 50–100%. The most common sources of whole-grain rye were 100% whole-grain rolled rye flakes for breakfast and rye bread with 60% whole-grain rye for lunch. Additionally, other rye cereals and rye pasta were consumed regularly. Twelve months after baseline (six months after end of intervention), the FFQ reported whole-grain rye intake was continued at a similar level as reported after 6 months of intervention. The FFQ reported rye intake was lower than the intake reported in the rye diaries since only whole-grain rye bread and crispbread, not cereals, pasta etc., was included in all three FFQs enabling a comparison only based on the baseline version (Table 3).

**Table 3:** Reported whole-grain rye intake by Food Frequency Questionnaire (FFQ) for intervention and control group, and rye diary and physical activity for the intervention group only, as well as compliance markers for the intervention and control group, respectively – the NILS feasibility study

	Intervention group (n = 14)						Control group (n = 7)						Difference between groups (95% CI)
	Baseline	3 months	6 months		12 months		Baseline	3 months	6 months		12 months		Baseline–6 months
	*Mean (SD)*	*Mean (SD)*	*Mean (SD)*	*Mean (SD)*	*Mean (SD)*	*Mean (SD)*	*Mean (SD)*	*Mean (SD)*	*Mean (SD)*	*Mean (SD)*	*Mean (SD)*	*Mean (SD)*	*Difference (95% CI)*
Whole-grain rye intake as reported in FFQ (g/day)	61 (24)	N/A	91^a^ (33)	182^b^ (42)	87^a,d^ (32)	200^b,d^ (130)	57 (26)	N/A	56^a^ (23)	90^b^ (20)	54^a,e^ (19)	128^b,e^ (49)	
Compliance markers of whole-grain intake													
*Total plasma alkylresorcinols (nmol/L)*	109 (98)	197 (87)	211 (144)		139^d^ (76)		74 (39)	108 (66)	102 (74)		109 (55)		93 (-30;215)
*C17:C21 plasma alkylresorcinols ratio*	0.39 (0.19)	0.43 (0.17)	0.42 (0.20)		0.49^d^ (0.21)		0.30 (0.10)	0.32 (0.10)	0.29 (0.05)		0.58 (0.25)		0.08 (-0.07;0.22)
	Intervention group (n = 14 for whole-grain intake and n = 12 for physical activity measures)						Control group No assessment of whole-grain intake from diaries or physical activity done for the control group						
	Baseline–3 months		3–6 months		12 months								
	*Mean (SD)*		*Mean (SD)*										
Whole-grain rye intake as reported in diary (g/day)	146 (19)		125 (40)		N/A								
	*Median (P5;P95)*		*Median (P5;P95)*										
Physical activity (min/week)													
*Above 70% of max - based on heart rate monitor*	91 (17, 193)		66 (13, 259)		N/A								
*Total - based on heart rate monitor*	220 (31, 338)		150 (66, 319)		N/A								
*Total - based on heart rate monitor + diary*	285 (58, 452)		225 (77, 355)		N/A								

*SD* standard deviation, *FFQ* food frequency questionnaire, *N/A* not applicable (not recorded), *P5* 5th percentile, *P95* 95th percentile

^a^Rye bread and rye crispbread

^b^Rye bread, rye crispbread, rye breakfast cereals, rye pasta, rye kernels, rye biscuits and *øllebrød*

^d^n = 12 in intervention group

^e^n = 6 in control group

Both total alkylresorcinols and C17:0/C21:0 ratio, markers of compliance, increased and especially between baseline and 3 months. At end of follow-up, total alkylresorcinols concentration was lower, but the mean C17:0/C21:0 ratio was 0.49 (SD: 0.21) indicating a continued high proportion of whole-grain rye to whole-grain wheat intake (Table 3). There was a tendency of a higher mean change in the intervention group compared to the control group for total alkylresorcinols (mean change from baseline to 6 months: 93 nmol/L, 95% CI: -30–215) and for the C17:0/C21:0 ratio (mean change from baseline to 6 months: 0.08, 95% CI: -0.07–0.22). The control group did not change their habitual intake of whole-grain rye from baseline to the end of the intervention period, as reported in the FFQs. This was supported by the C17:0/C21:0 ratio, which did not show any increase over the intervention period. Conversely, the concentration of total alkylresorcinols, reflecting the total whole-grain wheat and rye intake, increased in the control group during the intervention. However, the C17:0/C21:0 ratio decreased, suggesting that the proportion of whole-grain wheat intake must have increased. At 12 months (end of follow-up and 6 months after the intervention ended), the C17:0/C21:0 ratio was doubled in the control group, but no changes in the FFQ reported rye intake was seen.

### Physical activity

**Fig. 2:**
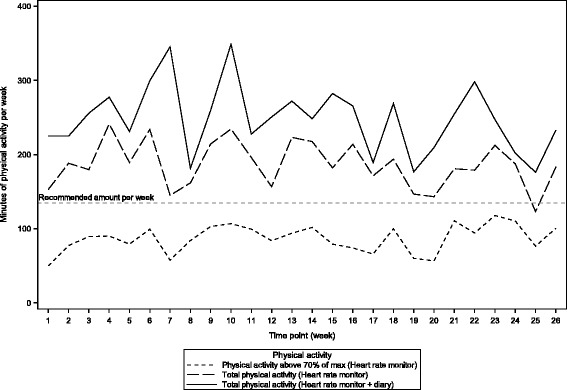
Physical activity in the intervention group, by heart rate monitors and diaries, for 26 weeks – the NILS feasibility study

Of the 14 men in the intervention group, 12 provided physical activity data from their heart rate monitors. Of these, there were missing or unrecorded data for a period of 2–3 weeks of the 26 weeks for four participants. According to the heart rate monitor data (Fig. 2), a median level of 91 (P5-P95: 17, 193) min/week of vigorous activity for the first 3 months and 66 (P5-P95; 13, 259) min/week for the last 3 months was performed. The total amount of physical activity, assessed both by heart rate monitor and the physical activity diaries, was 285 (P5-P95: 58, 452) min/week per week for the first 3 months and 225 (P5-P95: 77, 355) min/week for the last 3 months (Table 3).

### Effects of the intervention

**Fig. 3:**
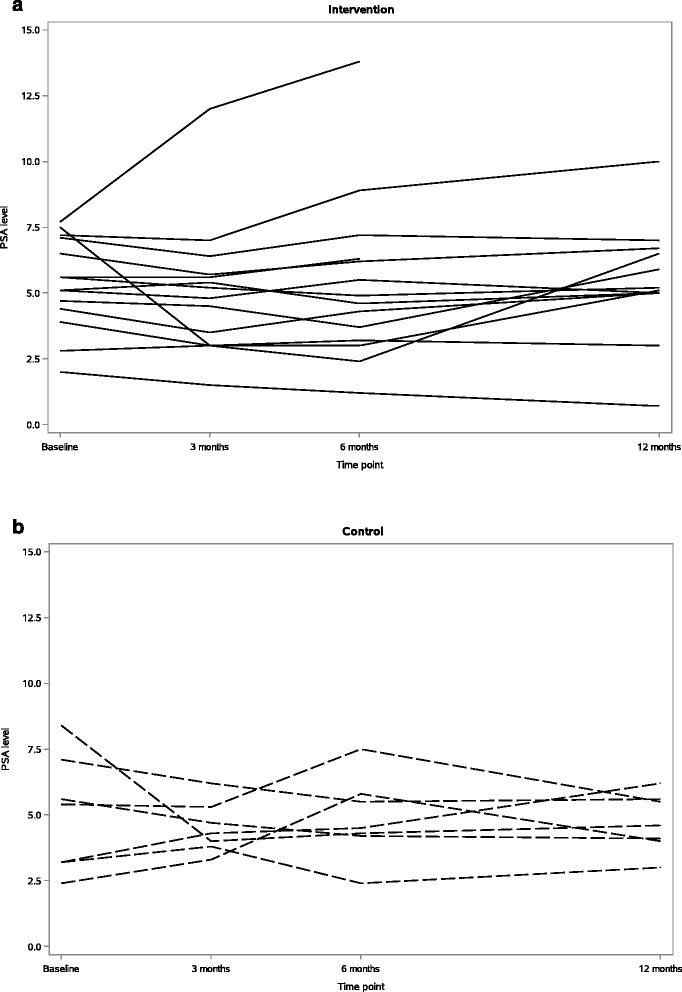
PSA levels at baseline and 3, 6 and 12 months after baseline in the intervention (**a**) and the control (**b**) group in the NILS feasibility study

Aerobic fitness evaluated by VO_2_ peak increased by 2.8 (95% CI: 0.1, 5.4) ml O_2_/min/kg in the intervention group compared to the control group after 6 months. There was no statistically significant difference in mean change of body composition, cardio-metabolic outcomes, or PSA levels between the intervention and the control group after 6 months (Table 4). However, there was a tendency for a decrease in waist circumference and plasma concentrations of cholesterols in the intervention group compared to the control group. The difference in mean change for waist circumference was -3 cm (95% CI: -7, 1). For LDL cholesterol and total cholesterol, mean changes of -0.3 mmol/l (95% CI: -0.7, 0.0) and -0.4 mmol/l (95% CI: -0.8, 0.1) respectively, were observed. In general, cardio-metabolic outcomes and PSA levels were similar from end of intervention to end of follow-up, and the increased aerobic fitness level was also maintained 6 months after end of intervention. The PSA levels did not change in any clear direction according to the intervention, as shown in Fig. 3.

**Table 4:** Outcome measures (means with standard deviations) in intervention and control arm participants of the NILS feasibility study at baseline, 3 and 6 months after baseline, and end of follow-up (12 months after baseline)

	Intervention (n = 14)				Control (n = 7)				Difference between groups (95% CI)
	Baseline	3 months	6 months	12 months^a^	Baseline	3 months	6 months	12 months	Baseline–6 months
	*Mean (SD)*	*Mean (SD)*	*Mean (SD)*	*Mean (SD)*	*Mean (SD)*	*Mean (SD)*	*Mean (SD)*	*Mean (SD)*	*Difference (95% CI)*
Physical fitness									
Fat-free mass (kg)	65 (7)	66 (9)	65 (8)	64 (8)	64 (6)	N/A	64 (6)	63 (6)	-0.2 (-1.5, 1.1)
Fat mass (%)	25 (6)	24 (5)	24 (6)	23 (5)	23 (4)	N/A	23 (5)	23 (5)	-0.5 (-2.4, 1.5)
VO^2^ peak (ml O^2^/min/kg)	29 (7)	32 (7)^b^	32 (7)	33 (6)^c^	26 (5)	N/A	27 (5)	28 (6)^d^	2.8 (0.1, 5.4)
Cardio-metabolic outcomes									
BMI (kg/m^2^)	26.0 (3.4)	25.8 (3.3)	25.7 (3.4)	25.0 (3.0)	25.6 (2.5)	25.6 (2.9)	25.4 (2.6)	25.4 (2.6)	-0.2 (-0.9, 0.6)
Waist circumference (cm)	102 (15)	99 (14)	100 (13)	95 (10)	98 (6)	N/A	99 (5)	97 (4)	-3 (-7, 1)
HDL cholesterol (mmol/L)	1.3 (0.2)	1.3 (0.3)	1.3 (0.2)	1.4 (0.3)	1.3 (0.2)	1.3 (0.1)	1.4 (0.2)	1.5 (0.2)	-0.0 (-0.2, 0.1)
LDL cholesterol (mmol/L)	2.8 (0.7)	2.8 (0.7)	2.7 (0.7)	2.8 (0.6)	2.9 (0.6)	2.8 (0.7)	3.1 (0.7)	3.0 (0.6)	-0.3 (-0.7, 0.0)
Total cholesterol (mmol/L)	5.1 (0.7)	5.1 (0.7)	5.0 (0.8)	5.2 (0.7)	5.3 (0.8)	5.3 (1.0)	5.6 (1.0)	5.6 (0.9)	-0.4 (-0.8, 0.1)
Triglycerides (mmol/L)	1.3 (0.4)	1.3 (0.4)	1.4 (0.6)	1.3 (0.5)	1.5 (0.6)	1.4 (0.4)	1.3 (0.5)	1.2 (0.3)	0.3 (-0.1, 0.7)
Fasting plasma glucose (mmol/L)	5.8 (0.6)	5.8 (0.4)	5.8 (0.5)	5.7 (0.4)	6.1 (0.4)	6.1 (0.3)	6.0 (0.2)	5.8 (0.3)	-0.1 (-0.5, 0.3)
Insulin (mmol/L)	11.6 (6.5)	10.2 (6.5)	11.4 (7.7)	8.0 (4.1)	11.8 (6.8)	15.1 (11.9)	9.7 (2.7)	10.2 (8.1)	1.8 (-2.9, 6.5)
Prostate cancer progression									
Prostate-specific antigen (PSA) (ng/ml)	5.4 (1.7)	5.0 (2.5)	5.4 (3.2)	5.4 (2.2)	5.0 (2.2)	4.5 (1.0)	4.9 (1.6)	4.7 (1.1)	0.2 (-2.1, 2.6)

Difference between group means adjusted for baseline level of outcome (95% CI)

*SD* standard deviation, *N/A* not applicable (not recorded), *VO* ^*2*^ *peak* peak oxygen consumption per unit time, *BMI* body mass index, *HDL* high-density lipoprotein, *LDL* low-density lipoprotein

^a^End of follow-up: n = 12 in intervention group

^b^n = 13 (one missing due to heart problems)

^c^n = 11

^d^n = 6

Information on side effects from the intervention was not systematically collected for the study, but some of the participants discussed problems with increased flatulence and stomach pain with the study dietician.

## Discussion

In this feasibility study, men diagnosed with non-aggressive prostate cancer managed to consume a high amount of whole-grain rye and to engage in vigorous physical activity during a 6-month intervention period, and thus the study found that it was feasible to complete such an intervention. Aerobic fitness increased significantly in the intervention group compared to the control group and this was maintained 6 months after end of intervention. No significant effects were found on cardio-metabolic outcomes or PSA levels between the intervention and control group, which was not surprising with the limited power due to the small sample size of the feasibility study. However, tendencies for lower waist circumference and cholesterol levels were observed in the intervention group compared to the control group. A full-scale intervention study is needed to investigate the health effects of the intervention further.

### Strengths and limitations

The study participants were selected by strict criteria, ensuring a homogenous group, and with the randomised design, the risk of potential confounding or uneven distribution hereof is minimised.

The regular follow-up meetings, physical examinations, diaries, monitors and biomarkers made it possible to evaluate compliance to the intervention during the entire study period. The compliance was generally good, however, the participants failed to fully meet the targets of 170 g of whole-grain rye per day and 3 × 45 minutes of vigorous physical activity per week on average over the 6-month intervention period. Five out of twenty-six (20%) randomised participants did not complete the 6-month intervention, which was as expected. Furthermore, two participants out before the 12-month follow-up. We do not suspect drop-out to have influenced our results, as the explanations seems unrelated to the intervention. Drop-out due to prostate cancer progression was, however, reported only for participants in the intervention group, but since the intervention group included two times the number in the control group, this could be purely due to chance. Furthermore, two of the participants who experienced prostate cancer progression and dropped out, did so during the 6 months after the active intervention period, making an association to the intervention itself unlikely. In future studies, it is important to retain the participants that drop out in the study, thus enabling intention-to-treat analyses.

The NILS study was designed to test the feasibility of a lifestyle intervention with whole-grain rye and vigorous physical activity in a group of men with prostate cancer considered to be non-aggressive. The sample size of 21 participants with complete data for the 6-month intervention was small, and statistical tests therefore had limited power. The focus was accordingly on the implementation of whole-grain rye and physical activity in the daily life of men with prostate cancer. Blinding to the intervention/control arm was not possible in this study as no corresponding treatment was offered to the control group. This is a general problem in both whole-grain and exercise interventions as taste and appearance of cereals (and especially rye) are difficult to mask, as is introducing placebo exercise. The NILS study did draw attention in the media, and it is likely that this also reached men in the control group. Hence, it is possible that participants in the control group changed their habits by, for example, increased whole-grain rye intake and physical activity. The alkylresorcinol C17:0/C21:0 ratio, however, did not increase in the control group during the intervention period and therefore we do not suspect whole-grain rye intake to have increased in this group. Data to quantify physical activity level of participants in the control group were not collected during the intervention period. However, VO_2_ peak did not increase notably, suggesting no apparent change in physical activity pattern in the control group.

### Feasibility of a high whole-grain rye intake during 6 months

We addressed compliance with the intervention by independent measures, i.e. self-reported whole-grain rye intake and with biomarkers reflecting total whole-grain wheat and rye intake and the proportion of whole-grain rye to total whole-grain wheat and rye intake: total alkylresorcinols and C17:0/C21:0 ratio. The high reported intakes of whole-grain rye were supported by high alkylresorcinols concentrations during the study period and with a high C17:0/C21:0 ratio, showing that the dietary intervention was successful in regards to whole-grain rye intake. The participants, however, decreased their intake during the last 3 months of the intervention compared to the first 3 months, which could indicate that the aim of 170 g of whole-grain rye per day was too high for a realistic long-term intake during 6 months. Even though Danish men have a generally high whole-grain intake, many participants complained about stomach pain and increased flatulence especially at the beginning of the intervention as the whole-grain rye intake increased significantly. If participants complained about such side effects, they were instructed to stepwise increase the amount of whole-grain rye per day during approximately 2 weeks before reaching the full amount. Reduced intake of fibre-rich fruits and vegetables for this run-in period was also suggested by the study dietician to avoid problems. For a potential future full-scale study, this is an important consideration as stomach pain and increased flatulence can lead to unnecessary drop-out or non-compliance with target intake and a run-in period could be introduced to avoid such problems. Furthermore, to ensure compliance, it may also be relevant to follow the participants even closer e.g. using modern technology (e.g. mobile applications) and to involve the spouses of the prostate cancer patients [28].

### Feasibility of implementing vigorous physical activity during 6 months

The physical activity part of the intervention was evaluated by heart rate monitors and physical activity diaries. The aim of 3 × 45 minutes of vigorous physical activity per week was not achieved (Fig. 2). However, the total amount of reported physical activity was of more than 4 hours per week, of which at least 1–1.5 hours was completed at a vigorous level (70% of maximal heart rate) indicating an acceptable compliance for this training intervention, despite the lack of supervised exercise sessions. The physical fitness level at baseline for the study participants corresponded to a low physical fitness level for men in the age group between 50–70 years compared to results from a subsample from the Danish Health Examination Survey 2007–2008 [29].

The observed improvement in VO_2_ peak in the intervention group of around 3 ml O_2_/min/kg is in accordance with similar studies with participants in the same age group and with baseline fitness levels in the same low range (25–28 ml O_2_/kg/min), but with physical activity interventions of 12 weeks [30] and 6 months [31] durations, respectively. Whether the improved fitness level obtained in NILS is of clinical relevance is important to consider in future initiatives. An increase of 3.5 ml O_2_/min/kg (equal to 1 MET) was in a meta-analysis associated with 13–15% lower risk of all-cause mortality and cardiovascular disease in healthy men and women [32]. The impact on prostate cancer progression is of course more relevant for this study and this has been investigated in prospective study designs where moderate-vigorous physical activity was associated with lower risks (44–61%) of prostate cancer-specific mortality [17, 19] and lower rate of progression [18].

### Effects of the intervention

Evaluation of the effect of the intervention on cardio-metabolic outcomes and PSA levels was a secondary aim of this feasibility study and the power was not sufficient for statistical testing. Individual change in PSA levels during the intervention and 6 months after, showed large variation with no clear pattern, which might not have been the case with a larger sample size. However, we regard the implementation of whole-grain rye and physical activity as feasible, and further positive tendencies on aerobic fitness, waist circumference and cholesterols were observed. Therefore, a full-scale study should be encouraged for further exploration of the effects of a feasible lifestyle prevention strategy on cardio-metabolic outcomes and prostate cancer disease progression.

## Conclusions

Men with prostate cancer managed to consume large amounts of whole-grain rye, reported in whole-grain rye diaries and confirmed by whole-grain rye compliance biomarkers. The level of vigorous physical activity increased during the intervention and as a result, aerobic fitness improved among participants in the intervention group. There was no statistical difference between the intervention and control group on cardio-metabolic outcomes or PSA levels, but this was as expected as the power was low due to the feasibility focus of the study. However, we find that the successful lifestyle implementations of whole-grain rye and physical activity encourage a full-scale study powered to investigate effects on prostate cancer progression specifically.
